# Effects of Pretreatment with a Combination of Melatonin and Electroacupuncture in a Rat Model of Transient Focal Cerebral Ischemia

**DOI:** 10.1155/2013/953162

**Published:** 2013-11-20

**Authors:** Lingguang Liu, R. T. F. Cheung

**Affiliations:** Department of Medicine, Li Ka Shing Faculty of Medicine, University of Hong Kong, Hong Kong

## Abstract

Both melatonin and electroacupuncture (EA) have been suggested to be effective treatments against stroke. However, it is unknown whether a combination of these two therapies could be beneficial against transient focal cerebral ischemia. The present study investigated the effects of pretreatment of a combination of melatonin and EA in a rat model of transient middle cerebral artery occlusion (MCAO). After pretreatment of melatonin plus EA (MEA), transient MCAO was induced for 90 minutes in male Sprague-Dawley (SD) rats. The neurological deficit score, brain infarct volume, cerebral edema ratio, neuronal inflammation, and apoptosis were evaluated 24 hours after transient MCAO. The expression of related inflammatory and apoptotic mediators in the brain was also investigated. The results showed that MEA improved neurological outcome, reduced brain infarct volume, and inhibited neuronal inflammation as well as apoptosis 24 hours after transient MCAO. The beneficial effects may derive from downregulation of proinflammatory and proapoptotic mediators and upregulation of antiapoptotic mediators. Thus, these results suggest a preventive effect of pretreatment of MEA on transient focal cerebral ischemia.

## 1. Introduction

Stroke is a serious cerebral vascular event with increasing prevalence worldwide especially in the society with aging of the population, and ischemic stroke is the most common type. Intravenous thrombolysis using recombinant tissue plasminogen activator (rtPA) is the only approved treatment [1]. Many neuroprotectants have been investigated; they were effective in animals but not in stroke patients [2–4]. Owing to the narrow therapeutic time window [5, 6] and a substantial risk of hemorrhagic complications, clinical use of rtPA is limited to a small number of stroke patients [7]. Thus, broader attention to integrated therapeutics has been advocated repeatedly for treating ischemic stroke to increase the chance of success [4, 8, 9]. Moreover, most of the current therapies are focused on posttreatment after cerebral ischemia. However, accumulating lines of evidence have demonstrated the efficacy of pretreatment therapies which could induce neuroprotection against cerebral ischemic injury.

Melatonin is a potent antioxidant and free radical scavenger with few toxicological effects in animals and humans [10]. An adequate amount of evidence indicates its effective protection against stroke in different models and animal species [11–16]. Although the beneficial effect of melatonin as well as its related mechanisms can be further investigated, some clinical scientists have suggested that melatonin, combined with other neuroprotectants or proven therapies, may enhance the treatment effects or extend the therapeutic time window. Moreover, it is worthy of conducting phase II or III clinical trials of melatonin in stroke patients [11].

Acupuncture has been widely applied to stroke patients in East Asia for centuries. It is easy to manipulate, economical and safe. EA is a combination of traditional acupuncture (manual acupuncture) and electrical stimulation. It is believed to enhance the efficacy of acupuncture and is currently used to treat various kinds of illnesses [17, 18]. Though the research in this field using western scientific methods is still in the very beginning, increasing clinical and experimental publications have provided physiological rather than metaphysical evidence to confirm and explain both the phenomena and mechanisms of acupuncture [18–22]. Nevertheless, the beneficial effects of acupuncture on stroke recovery remain controversial. This may be due to poor experimental design, inappropriate controls, and small sample size [23].

In traditional Chinese medical theory, acupuncturing at the acupoints along the meridian of Yangming (stomach meridian) is especially efficient for the treatment of flaccid paralysis. Zusanli (ST 36) is the classical acupoint recorded in the ancient Chinese medical literature for thousands of years and has been frequently investigated in scientific study for treating stroke. Moreover, the literature review shows that ST 36 and xiajuxu (ST 39) are the mostly investigated acupoints including animal and clinical studies [24, 25]. Their beneficial effects on ischemic stroke have also been elucidated and reported. In the present study, both ST 36 and ST 39 were chosen for EA treatment.

Evidence from animal research in acute cerebral ischemia shows that combinations of neuroprotectants might be more efficacious than the single agent given alone. Both melatonin and EA have been suggested to be effective treatments against cerebral ischemia. However, it is unknown whether a combination of these two therapies could be beneficial against focal cerebral ischemia.

There are increasing lines of evidence that pretreatment of neuroprotectants effectively improves neurological outcome and interferes with mechanisms of brain injury [26, 27]. In the present study, pretreatment of MEA was conducted to examine whether MEA could exert neuroprotection against transient cerebral ischemia and which mechanisms are behind it.

## 2. Materials and Methods

### 2.1. Animals and Surgical Procedures

Adult male SD rats, weighing between 250 and 280 g, were obtained from the Laboratory Animal Unit, The University of Hong Kong, and kept under 12 hours light/12 hours dark conditions. The experiments were performed according to the institutional regulation and guidelines approved by the Committee on the Use of Live Animals in Teaching and Research (CULATR), The University of Hong Kong.

Transient focal cerebral ischemia was induced using right-sided transient endovascular MCAO [28–30] with reperfusion. In brief, the right common carotid artery (CCA), right external carotid artery (ECA), and right internal carotid artery (ICA) were exposed through a midline cervical incision. With the right ECA dissected free and its distal branches coagulated (Bipolar Electric Coagulation, GN60, Aesculap AG and Co., Tuttlingen, Germany), a 5-o silk suture was loosely tied around the ECA stump, and microclips were temporarily placed at both the right CCA and right ICA. A piece of silicone coated 4-o (5 mm coating length with 0.35–0.37 mm diameter) nylon suture (Doccol Corporation, Redlands, CA; Cat no. 4037) was inserted into the lumen of right ECA stump, and the 5-o silk suture was gently tightened to prevent bleeding. Next the nylon suture was gently advanced through right ICA into the right anterior cerebral artery (ACA) to occlude the right middle cerebral artery (MCA) at its origin. The rats were subjected to 90 minutes of focal cerebral ischemia, and then the silicon coated 4-o nylon suture was carefully withdrawn to permit reperfusion. The wounds were closed using 4-o nylon sutures, and the rats were allowed to fully recover from the anesthesia before returning to their cages.

The focal cerebral ischemia was confirmed by obvious changes of regional cerebral blood flow (rCBF) on laser Doppler flow meter (MBF3D, Moor instruments, Ltd., Devon, UK). A burr hole of 2 mm diameter was made on the right side of skull at 5 mm lateral and 2 mm posterior to the bregma with the aid of a stereotaxic device (SR-6N; Narishige Scientific Instrument Laboratory, Tokyo, Japan). Next the laser probe was glued onto the burr hole. Steady-state baseline values were documented before induction of cerebral ischemia, and all rCBF were normalized and expressed as percentages of baseline values.

### 2.2. Experimental Groups

Rats were assigned to one of two groups: control group and MEA group.

1. Control group: the vehicle, normal saline containing 3% dimethyl sulfoxide (DMSO, Sigma-Aldrich, St. Louis, MO, USA), was given via an intraperitoneal (i.p.) injection 30 minutes before transient MCAO. Sham EA treatment was given once per day and started 6 days before transient MCAO. During the sham EA treatment, rats were anesthetized with an i.p. injection of sodium pentobarbital at a dose of 40 mg/kg without real EA stimulation.
2. MEA group: a single dose of melatonin (10 mg/kg) was given via an i.p. injection 30 minutes before transient MCAO. Whilst under anesthesia, EA treatment was given once per day and started 6 days before transient MCAO. Melatonin (Sigma-Aldrich) was dissolved in 1 mL of normal saline containing 3% DMSO.

**Figure 1:**
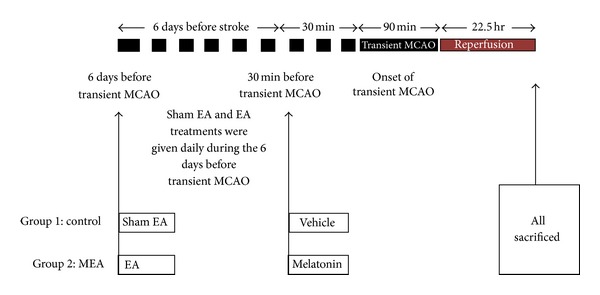
Schematic overview of the experimental procedures in a rat model of transient MCAO. Two groups are included in the experiment. The horizontal line represents time. A single i.p. injection of the vehicle and melatonin was given 30 minutes before transient MCAO in control and MEA groups, respectively. Whilst the rats were under anesthesia, sham and real EA were given daily 6 days before transient MCAO in the sham EA group and EA group, respectively. All the rats were killed 24 hours after transient MCAO for determination of brain infarct volume, cerebral edema ration and tissue processing.

A schematic overview of the experimental procedures was summarized (Figure 1). All the rats were sacrificed 24 hours after transient MCAO for determination of brain infarct volume, cerebral edema ratio, and tissue processing. Neurological outcome of rats was evaluated before sacrifice.

### 2.3. Manipulation of EA Treatment

**Figure 2:**
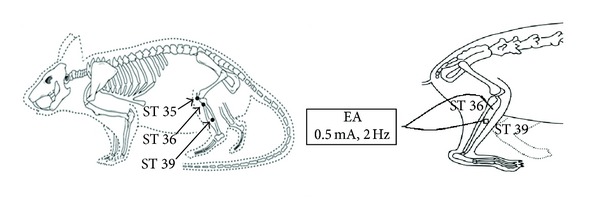
Diagram of the acupoints location and acupuncture manipulation in the rat. ST 35, dubi, is located at the depression lateral to the patella ligament; ST 36, zusanli, is located at proximal one-fifth point on the line from ST 35 to the anterior side of ankle crease; ST 39, xiajuxu, is located at proximal 3 fifths point on the line from ST 35 to the anterior side of ankle crease.

A thirty-minute real or sham EA treatment was applied each time at different time points (daily administration during the 6 days before transient MCAO) with a Han's acupoint nerve stimulator (HANS-200, Jisheng Medical Science and Technology Co., Ltd., Nanjing, China). The stimulation intensity was 0.5 mA, and the stimulation frequency was 2 Hz. Four stainless steel acupuncture needles (0.25 mm in diameter, 25 mm in length; Huatuo, Suzhou Medical Instruments Factory, Suzhou, China) were inserted bilaterally with a 4 mm depth into two acupoints, ST 36 and ST 39. A Han's acupoint stimulator was connected to the inserted acupuncture needles. The location of these two acupoints was based on the transpositional acupoint system in a rat model [29], which was modified from a former animal transpositional acupoint system [30]. ST 36 is located on the line from dubi (ST 35) to the ankle crease of hind limb and is at proximal one-fifth point of the line. ST 39 is at proximal three-fifths point of the line between ST 35 and the ankle crease. ST 35 is located at the depression on the lateral side of the patella ligament of the hind leg (Figure 2).

### 2.4. Neurological Behavior Assessment

Neurological deficit scoring system (NDSS) test (Table 1) was used to quantify neurological behavior 24 hours after transient MCAO and was done by a blinded observer. NDSS test was adapted from a validated scoring system [31, 32]. The higher the score, the more severe was the injury. All the rats were trained before operation to be familiar with the testing environment.

### 2.5. Brain Infarct Volume and Cerebral Edema Ratio Measurement

Brain infarct volume of the right cerebral hemisphere was measured 24 hours after transient MCAO. Under deep anesthesia after an i.p. injection of pentobarbital at 100 mg/kg, the brains were removed and cut into coronal slices of 2 mm thick using a rodent brain matrix (World Precision Instruments, Inc., Sarasota, FL). After reaction with 2% 2,3,5-triphenyltetrazolium chloride (TTC; Sigma-Aldrich) for 20 minutes at 37°C, the slices of brain were fixed in 10% formalin (pH 7.4). Both sides of each slice were scanned for measurement of both hemisphere and infarct volume using a computer assisted image analysis system (Image J Ver. 1.36b, NIH, USA). Unstained areas represented the ischemic lesions. A cerebral edema ratio was calculated from the ratio of the volume of the right hemisphere to the left hemisphere. To compensate for the effect of brain swelling, the actual (corrected) infarct volume was calculated via dividing the volume of infarction by the edema ratio. Brain infarct volume was expressed as a percentage of the contralateral hemisphere [31, 32].

### 2.6. Histological Examination

At 24 hours after transient MCAO, rats were deeply anesthetized with an i.p. injection of pentobarbital at 100 mg/kg and transcardially perfused with 0.9% normal saline first and then with ice-cold 4% paraformaldehyde (Sigma-Aldrich) in 0.1 M phosphate buffer (PB; pH 7.5). Coronal brain sections at a thickness of 4 *μ*m were made from 2 mm anterior and 1 mm posterior to the bregma and stained with hematoxylin and eosin (HE). Terminal deoxynucleotidyl transferase dUTP nick end labeling (TUNEL) assay was also performed using an in situ cell death detection kit (Roche, Indianapolis, IN). Brain sections near bregma level 0 were chosen for quantification. The number of TUNEL-positive cells from five selected regions within the infarction and penumbra in the right cerebral hemisphere was counted, respectively. The number of positive cells was counted by a blinded investigator and expressed as number per square millimeter. The section slides were analyzed under light microscope (Axio Vision Control, Carl Zeiss, Munich, Germany).

### 2.7. Western Blot Analysis

Western blot analysis was used to determine the expression of inflammatory and apoptotic mediators. Rats from the two groups were decapitated 24 hours after transient MCAO. The infarct region (between 2 mm anterior and 1 mm posterior to the bregma) of the right cerebral hemisphere was dissected on ice. Samples were placed in radioimmune precipitation assay (RIPA) lysis buffer containing a protease inhibitor cocktail and a phosphatase inhibitor cocktail (Sigma-Aldrich). After sonication for 5 seconds, the lysate was kept on ice for 30 minutes. After 20 minutes centrifugation at 12,000 g, the supernatants were collected. The protein concentration was determined using a Bradford protein assay kit (Bio-Rad, Hercules, CA). Forty *μ*g of total protein was mixed with protein loading buffer and separated using 10–15% sodium dodecyl sulfate polyacrylamide gel electrophoresis (SDS-PAGE). After electrophoresis, the proteins were transferred to polyvinylidene difluoride (PVDF) membrane (Bio-Rad) at 4°C. After blocking nonspecific binding sites on the membrane with 5% nonfat milk in Tris-HCL-based buffered saline with 0.1% Tween 20 (TBST; Sigma-Aldrich) at pH 7.4 for 1 hour at room temperature, and the membranes were incubated at 4°C overnight with primary antibodies, including tumor necrosis factor-alpha (TNF-*α*) (1 : 200 dilution; Santa Cruz Biotechnology, Dallas, TX), cyclooxygenase 2 (COX-2) (1 : 500 dilution; Santa Cruz Biotechnology), B-cell lymphoma 2 (Bcl-2) (1 : 500 dilution; Santa Cruz Biotechnology), Bcl-2-associated X protein (Bax) (1 : 500 dilution; Santa Cruz Biotechnology), and *β*-actin (1 : 2000 dilution; Santa Cruz Biotechnology). After three washes (15 minutes per time) with TBST, the membranes were incubated with a horseradish peroxidase-conjugated goat secondary antibody (anti-mouse, 1 : 7000 dilution; Santa Cruz Biotechnology; or anti-rabbit, 1 : 7000 dilution; Santa Cruz Biotechnology) at room temperature for 1 hour. After three washes with TBST, the protein bands were visualized using advanced chemoluminescence (GE Healthcare Life Sciences, Hong Kong), recorded by GelDoc-2000 Imagine System (Bio-Rad), and the relative intensity of protein expression was quantified using Quantity One software (Bio-Rad).

### 2.8. Data Analysis

All the data were analyzed using SPSS (Window version 13.0; SPSS Inc., Chicago, IL) and expressed as mean ± SEM. One sample *t*-test was used to detect a significant change in rCBF data from the baseline value at different time points in each group. Data were compared using Student's *t*-test. *P* < 0.05 was used to infer statistical significance.

## 3. Results

### 3.1. Relative rCBF

During transient cerebral ischemia, the normalized rCBF was significantly decreased; there was no statistically significant difference at different time points within the same group. During reperfusion, the normalized rCBF returned towards baseline values; there was no statistically significant difference within the same group. No significant difference was observed between the control and MEA groups at the same time points during ischemia and reperfusion (*P* > 0.05), as shown in Tables 2, 3, and 4.

### 3.2. Effect of MEA on Neurological Deficit Score

**Figure 3:**
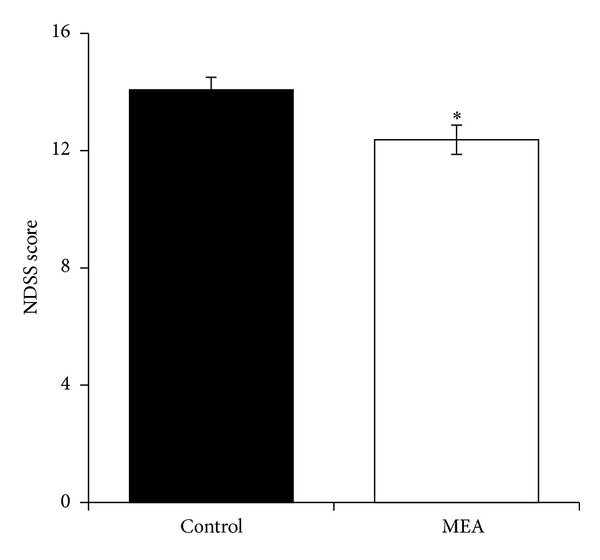
Neurological deficit scoring system (NDSS) score 24 hours after transient MCAO. Data are expressed as mean ± SEM (*n* = 14). *P < 0.05, compared with the control group. The neurological function was significantly improved by MEA pretreatment.

When compared with the control group (14.07 ± 0.43), neurological function was significantly improved by MEA pretreatment (12.37 ± 0.50) 24 hours after transient MCAO (*P* < 0.05), as shown in Figure 3.

### 3.3. Effect of MEA on Brain Infarct Volume and Cerebral Edema Ratio

**Figure 4:**
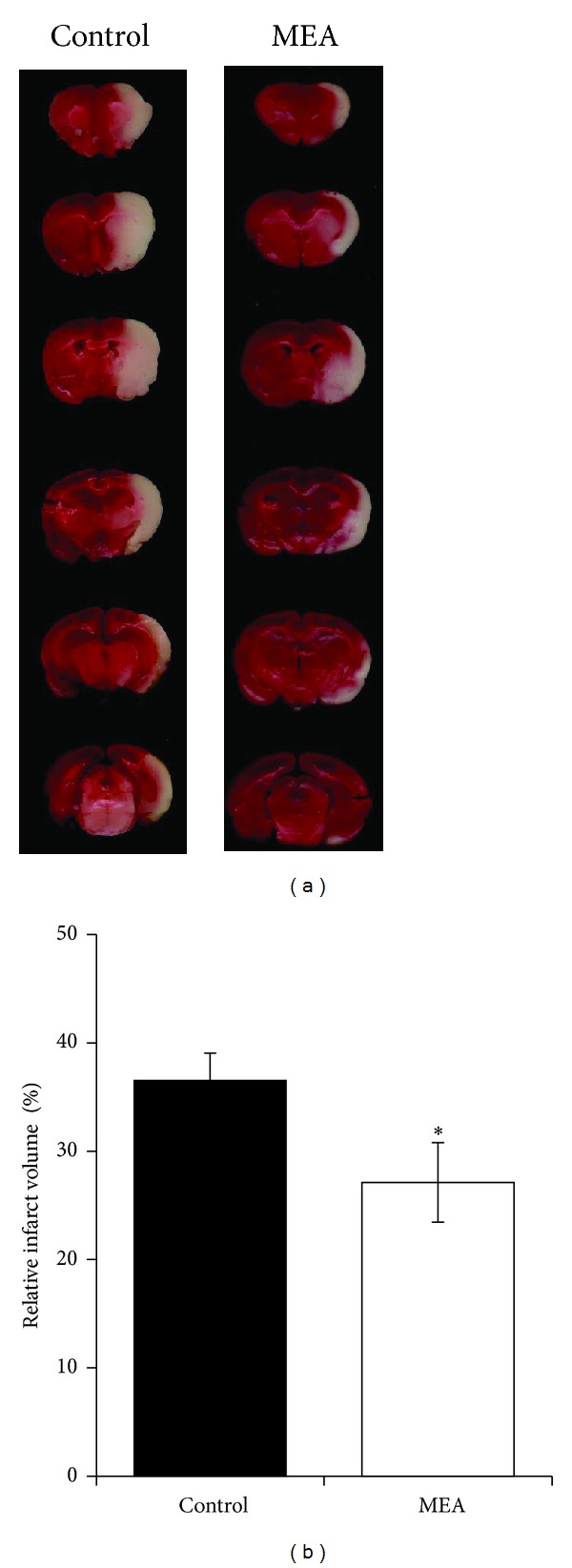
Brain infarct volume measurement 24 hours after transient MCAO. (a) Digital photographs of the 2 mm thick coronal brain slices between the bregma levels +4 mm (anterior) and −6 mm (posterior) in control and MEA groups 24 hours after right-sided endovascular transient MCAO. TTC reaction showed viable brain tissue in red and infarcted brain tissue in white. (b) Quantitative analyses of brain infarct volume 24 hours after transient MCAO. The data are expressed as percentage of the contralateral hemispheric volume (mean ± SEM, in %, *n* = 14). *Indicates the significant difference between the control group and MEA group (*P* < 0.05).

**Figure 5:**
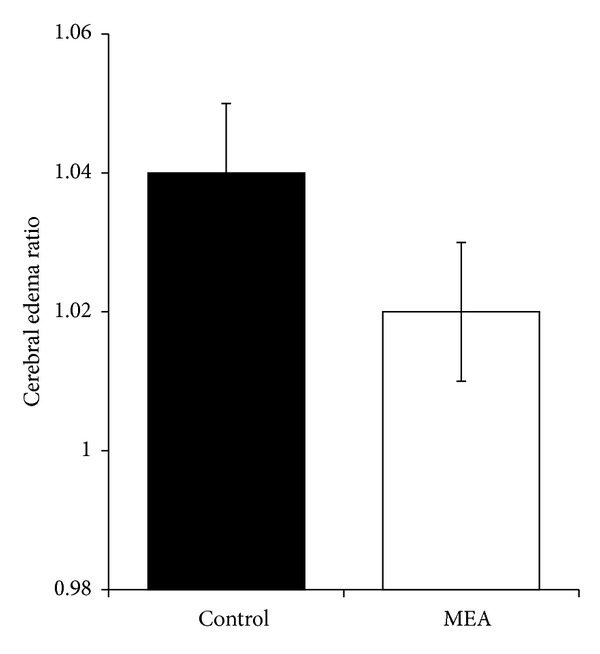
Data of cerebral edema ratio 24 hours after transient MCAO. Data are expressed as mean ± SEM (*n* = 14). Student's *t*-test reveals no significant difference in cerebral edema ratio 24 hours after transient MCAO between the control and MEA group (*P* > 0.05).

Figure 4(a) summarizes the representative brain slices after reaction with TTC 24 hours after transient MCAO. The right cerebral infarct was evident as the whitish region.  Figure 4(b) summarizes the computer assisted image analysis data revealing the relative brain infarct volumes in the two groups 24 hours after transient cerebral ischemia. The relative infarct volume was expressed as mean ± SEM. When compared with the control group (36.5 ± 2.6%), MEA pretreatment (27.1 ± 3.7%) significantly decreased the infarct volume by 25.7% (*P* < 0.05).  Figure 5 summarizes the data revealing the cerebral edema ratio in the two groups 24 hours after transient cerebral ischemia. There was no significant difference between the two groups (*P* > 0.05).

### 3.4. Effect of MEA on Histological Changes of Brain Inflammation 24 Hours after Transient MCAO

**Figure 6:**
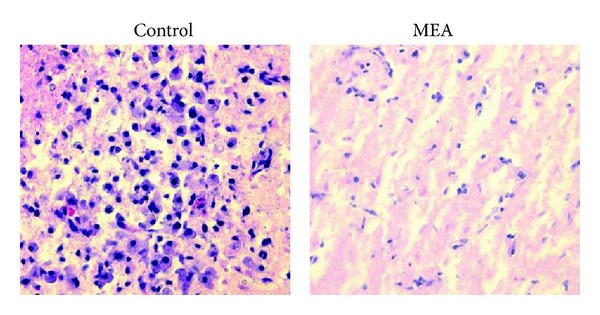
Photomicrographs of HE-stained brain sections near bregma level 0 24 hours after right-sided transient MCAO (magnification: 400x). In the control group, neutrophil infiltration was mainly present in the infarcted cortex. Neutrophil infiltration in the ischemic cerebral cortex was suppressed by the MEA pretreatment group 24 hours after transient MCAO.

Brain section near bregma level 0 stained with HE revealed the histological changes 24 hours after transient MCAO (Figure 6). In the control group, many necrotic neurons and infiltrated neutrophils were seen in the infarcted cortex after transient MCAO. In rats pretreated with MEA, neutrophil infiltration within the ischemic cerebral cortex was suppressed.

### 3.5. Effect of MEA on Histological Changes of Brain Apoptosis 24 Hours after Transient MCAO

**Figure 7:**
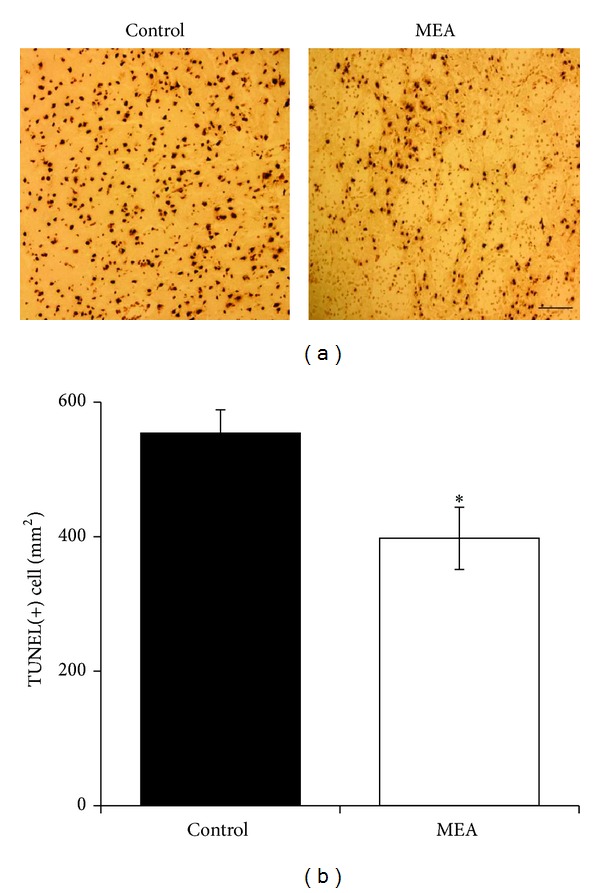
Apoptosis in the infarcted cortex of right ischemic cerebral hemisphere 24 hours after transient MCAO. (a) Representative images of TUNEL staining in the infarcted cortex of right ischemic cerebral hemisphere 24 hours after transient MCAO. The brown staining within the nuclei reveals TUNEL-positive cells. (b) Quantitative analysis of TUNEL-positive cells. Data are expressed as means ± SEM (*n* = 5). *P < 0.05, compared with the control group. The number of TUNEL-positive cells was significantly decreased by MEA pretreatment. Scale bar = 100 *μ*m.

**Figure 8:**
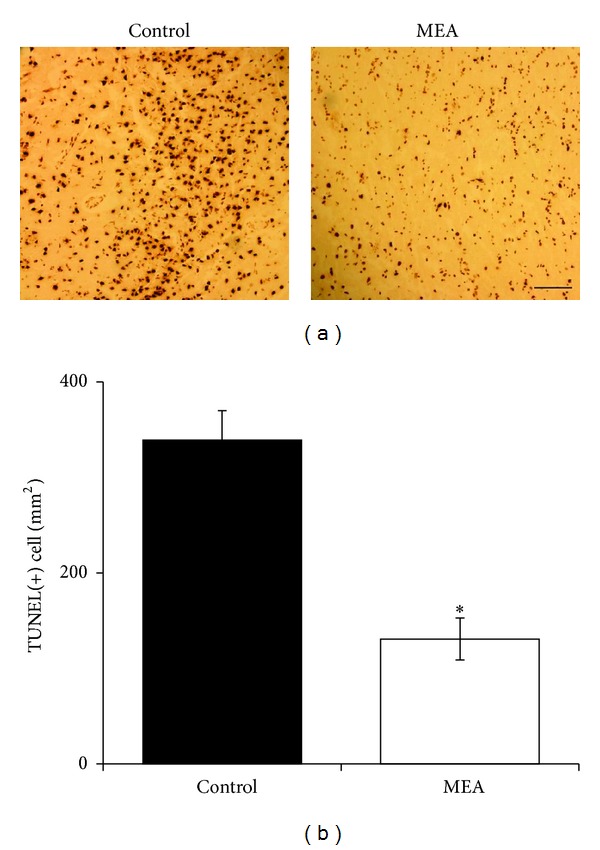
Apoptosis in the penumbra of right ischemic cerebral hemisphere 24 hours after transient MCAO. (a) Representative images of TUNEL staining in the penumbra of right ischemic cerebral hemisphere 24 hours after transient MCAO. The brown staining within the nuclei reveals TUNEL-positive cells. (b) Quantitative analysis of TUNEL-positive cells. Data are expressed as means ± SEM (*n* = 5). *P < 0.01, compared with the control group. The number of TUNEL-positive cells was significantly decreased by MEA pretreatment. Scale bar = 100 *μ*m.

Five regions within the cortex and penumbra were respectively selected for cell counting on the right cerebral hemisphere. Many TUNEL-positive cells were seen within the infarct and penumbral areas of the right cerebral hemisphere in the control group 24 hours after transient MCAO. In the MEA pretreatment group, increase in the number of TUNEL-positive cell within the ischemic cerebral cortex and penumbra was significantly reduced (Figures 7 and 8).

### 3.6. Effect of MEA on Protein Expression of Inflammatory Mediators 24 Hours after Transient MCAO

**Figure 9:**
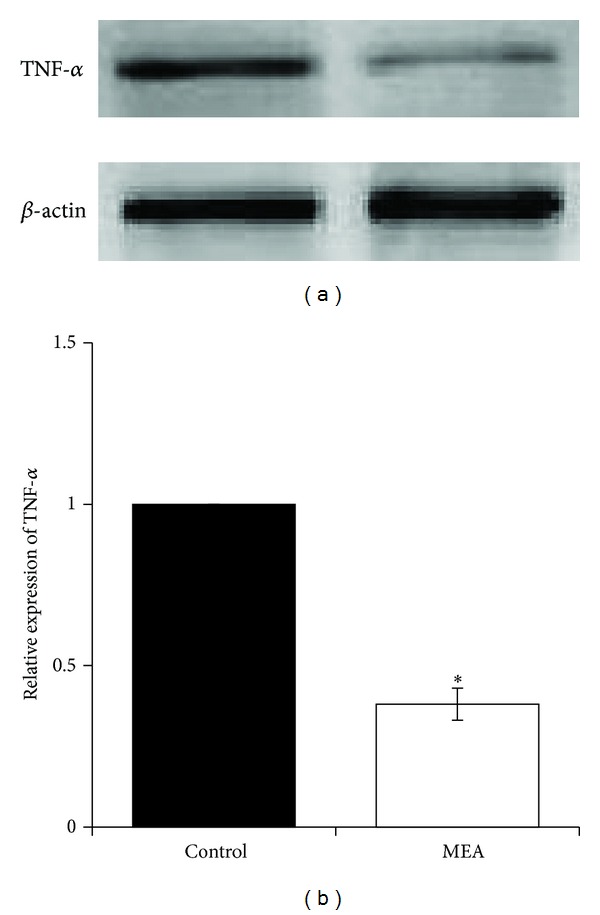
Protein expression of TNF-*α* 24 hours after transient MCAO in different groups. (a) Representative immunoblots of TNF-*α* in the right cerebral hemisphere of different groups 24 hours after transient MCAO. (b) Semiquantitative analysis of protein expression of TNF-*α*. Data are expressed as means ± SEM (*n* = 3). *P < 0.01, compared with the control group. The relative expression of TNF-*α* was significantly inhibited by MEA pretreatment.

**Figure 10:**
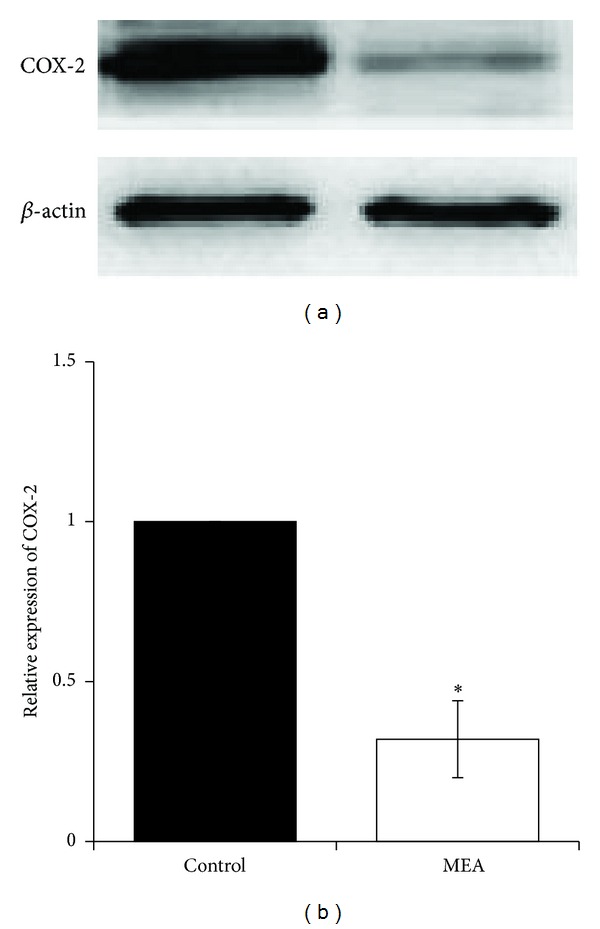
Protein expression of COX-2 24 hours after transient MCAO in different groups. (a) Representative immunoblots of COX-2 in the right cerebral hemisphere of different groups 24 hours after transient MCAO. (b) Semiquantitative analysis of protein expression of COX-2. Data are expressed as means ± SEM (*n* = 3). *P < 0.01, compared with the control group. The relative expression of COX-2 was significantly decreased by MEA pretreatment.

The expression of inflammatory mediators, including TNF-*α* (Figure 9) and COX-2 (Figure 10), was investigated using western blot analysis 24 hours after transient MCAO. Immunoblots of these inflammatory mediators were obtained from right cerebral hemisphere. When compared with the control group, MEA pretreatment significantly decreased the upregulated protein expression of TNF-*α* and COX-2 (*P* < 0.01).

### 3.7. Effect of MEA on Protein Expression of Bcl-2 Family Proteins 24 Hours after Transient MCAO

**Figure 11:**
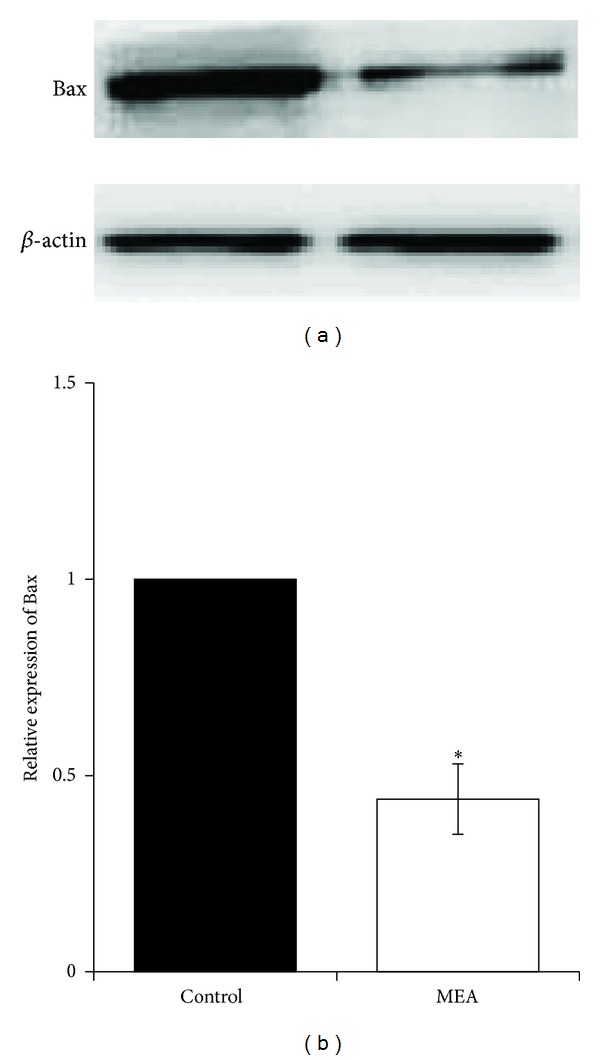
Protein expression of Bax 24 hours after transient MCAO in different groups. (a) Representative immunoblots of Bax in the right cerebral hemisphere of different groups 24 hours after transient MCAO. (b) Semiquantitative analysis of protein expression of Bax. Data are expressed as means ± SEM (*n* = 3). *P < 0.05, compared with the control group. The relative expression of Bax was significantly decreased by MEA pretreatment.

**Figure 12:**
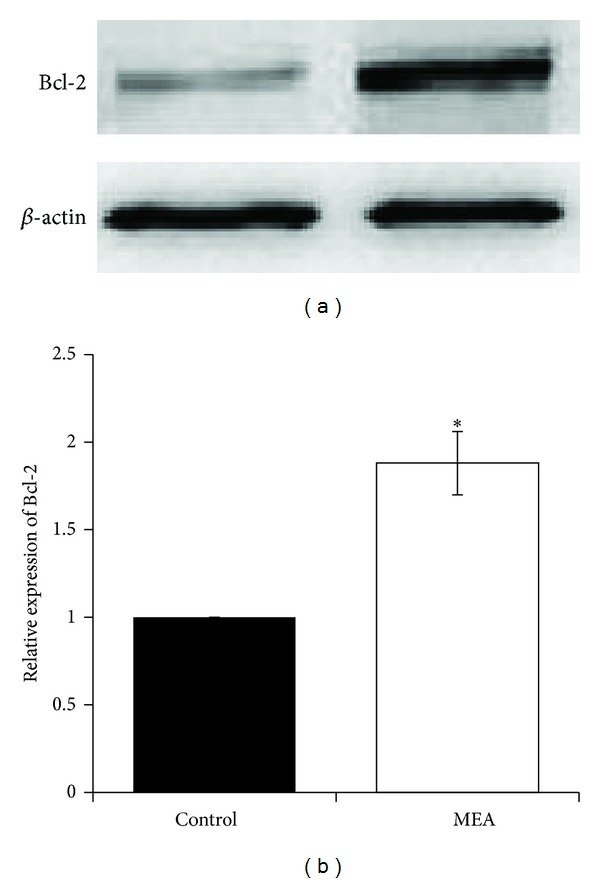
Protein expression of Bcl-2 24 hours after transient MCAO in different groups. (a) Representative immunoblots of Bcl-2 in the right cerebral hemisphere of different groups 24 hours after transient MCAO. (b) Semiquantitative analysis of protein expression of Bcl-2. Data are expressed as means ± SEM (*n* = 3). *P < 0.01, compared with the control group. The relative expression of Bcl-2 was significantly increased by MEA pretreatment.

The expression of Bax and Bcl-2 was investigated using western blot analysis (Figures 11 and 12) 24 hours after transient MCAO. Immunoblots of these proteins were obtained from right cerebral hemisphere. When compared with the control group, the relative protein expression of Bax was significantly decreased by MEA pretreatment (*P* < 0.05). The protein expression of Bcl-2 was significantly increased by MEA when compared with the control group (*P* < 0.01).

## 4. Discussion

Increasing evidence shows that pretreatment with various kinds of neuroprotectants induces beneficial effects against stroke in animal models; however, many of them have limitations and adverse effects that may prevent the clinical application in patients [33, 34]. Although it is suggested that therapeutics for prevention of first and recurrent stroke, such as blood pressure control and anti-thrombosis, are highly recommended in clinical practice [35, 36], some of these drugs, like antiplatelets and anticoagulants, have a risk of causing hemorrhage. Therefore, it is desirable to develop not only efficient but also safe strategies aiming at preventing cerebral ischemia as well as reducing ischemic injury. A previous study in our lab (unpublished data) has shown that posttreatment of MEA may induce neuroprotective effect in transient MCAO. The present study was performed to explore whether pretreatment of the combined therapy may prevent the brain from cerebral ischemic injury.

Firstly, the effect of MEA pretreatment on NDSS score, brain infarct volume, and cerebral edema ratio was investigated. The data showed that when compared with the control group, pretreatment of MEA significantly improved neurological functions 24 hours after transient MCAO. Meanwhile, MEA reduced the brain infarct volume by 25.7% 24 hours after transient MCAO. No significant changes in the cerebral edema ratio were observed. There were no significant differences in rCBF data during cerebral ischemia and reperfusion. 

Secondly, the effect of MEA pretreatment on histological and cellular inflammation after transient ischemic stroke was examined. HE staining of brain sections shows that many necrotic neurons and infiltrated neutrophils were seen in the infarcted cortex after transient MCAO. In rats pretreated with MEA, neutrophil infiltration within the ischemic cerebral cortex was suppressed. In addition, MEA pretreatment reduced the upregulated protein expression of two proinflammatory mediators, TNF-*α* and COX-2, in the ischemic right cerebral hemisphere 24 hours after transient MCAO. These results indicate the anti-inflammatory effects of MEA pretreatment of against transient MCAO.

Thirdly, the effect of MEA pretreatment on histological and cellular apoptosis after transient ischemic stroke was evaluated. In the present study, TUNEL staining of brain sections shows that there were many TUNEL-positive cells within the ischemic infarction and penumbra of the right cerebral hemisphere in the control group 24 hours after transient MCAO. The number of TUNEL-positive cells was significantly decreased in the same areas by MEA pretreatment 24 hours after transient MCAO.

Moreover, MEA pretreatment decreased the level of proapoptotic protein Bax and increased the level of antiapoptotic protein Bcl-2 24 hours after transient MCAO when compared to the control group. The present data suggest the antiapoptotic effect of MEA pretreatment in transient MCAO.

Previous research indicates that inflammatory response is induced in the cerebral infarct and its surrounding areas after cerebral ischemia [37]. Various proinflammatory mediators, such as TNF-*α* and COX-2, are upregulated after stroke. These mediators play critical roles in the process of neuronal survival following brain injury [38, 39]. Inhibition of TNF-*α* reduced the brain infarct volume and suppressed the inflammatory responses in a mouse model of transient cerebral ischemia [40]. Overexpression of COX-2 may exacerbate brain damage [41]. According to the present data, the neuroprotection with regard to neurological outcome and brain infarct volume may be partly due to the inhibitory effect of MEA on TNF-*α* and COX-2.

The damage resulting from cerebral ischemia is constituted by two principal zones: infarct core and ischemic penumbra. After ischemic stroke, neuronal apoptosis occurs mainly in the penumbral area [42]. In particular after transient focal ischemia, apoptosis may be a contributing factor to the final infarct volume [43]. According to the present data, the number of TUNEL-positive cells was significantly decreased in the penumbral area by MEA pretreatment 24 hours after transient MCAO. This finding may partly explain the result of infarct volume reduction after MEA pretreatment. In addition, Bax and Bcl-2 are suggested to be distinct regulators of apoptosis in the early stages of stroke [44]. Upregulation of Bax and downregulation of Bcl-2 are repeatedly observed in the penumbral area following cerebral ischemia [45]. Bax inhibition results in neuroprotection against stroke [46]. Decreased expression of Bcl-2 leads to increased oxidative stress [46, 47]. The present data indicate the antiapoptotic effect of MEA pretreatment via downregulation of Bax and upregulation of Bcl-2.

In conclusion, this study provides some preliminary data with regard to the effect of MEA pretreatment on transient focal cerebral ischemia. The present results indicate that MEA pretreatment may induce a neuroprotection against transient MCAO in terms of improved neurological function and decreased brain infarct volume. The beneficial effects are partly mediated by anti-inflammation and antiapoptosis.

## Figures and Tables

**Table 1:** Neurological deficit scoring system (NDSS) test.

		Points
Motor tests	*Spontaneous activity *	
	Approaches at least three sides of cage	0
	Approaches at least one rim but not all sides of cage	1
	Slight movement	2
	No movement	3
	*Floor walking *	
	Straight path	0
	Curvilinear path	1
	Walks only in circles	2
	No walking	3

Sensorimotor tests	*Raising rat by the tail *	
	Flexion of forelimb	1
	Flexion of hindlimb	1
	Thorax twisting	1
	*Left limbs placing task *	
	Forelimb	
	Normal performance	0
	Delayed (less than 2 seconds)	1
	Delayed (at least 5 seconds) and/or incomplete performance	2
	No performance	3
	Hindlimb	
	Normal performance	0
	Delayed (less than 2 seconds)	1
	Delayed (at least 5 seconds) and/or incomplete performance	2
	No performance	3

Beam balance tests	Walks on the beam	0
	Grasps side of beam	1
	Hugs the beam and one limb falls down from the beam	2
	Hugs the beam and two limbs fall down from the beam or spins on beam (>60 s)	3
	Attempts to balance on the beam but falls off (>40 s)	4
	Attempts to balance on the beam but falls off (>20 s)	5
	Falls off: with no attempt to balance or hang on the beam (<20 s)	6

Maximal number of point is 21. Points were awarded for inability of performing the tasks. The higher the score, the more severe is the injury.

**Table 2:** Normalized regional cerebral blood flow (rCBF, %) at different time points in relation to the onset of transient MCAO with MEA pretreatment in the study on neurological function and brain infarct volume in rats.

Group (number of rats)	Before MCAO	Onset of MCAO	30 min after MCAO	60 min after MCAO	Onset of reperfusion	30 min after reperfusion
Control (14)	100	23.3 ± 1.7	23.4 ± 1.6	25.2 ± 1.7	87.0 ± 3.3	99.4 ± 4.0
MEA (14)	100	24.8 ± 1.4	25.1 ± 1.8	26.8 ± 2.2	88.4 ± 4.2	94.1 ± 5.1

Data are expressed as mean ± SEM. Normalized rCBF dropped to less than 30% during transient MCAO and returned to more than 70% at the onset of reperfusion. There was no statistically significant difference between the control and MEA groups at the same time points (*P* > 0.05).

**Table 3:** Normalized regional cerebral blood flow (rCBF, %) at different time points in relation to the onset of transient MCAO with MEA pretreatment in the study on tissue apoptosis in the right cerebral hemisphere of the rats.

Group (number of rats)	Before MCAO	Onset of MCAO	30 min after MCAO	60 min after MCAO	Onset of reperfusion	30 min after reperfusion
Control (5)	100	24.6 ± 1.6	25.5 ± 1.5	27.2 ± 1.7	96.5 ± 6.2	98.0 ± 5.5
MEA (5)	100	23.3 ± 2.1	24.4 ± 1.9	26.2 ± 2.3	104.3 ± 6.8	102.1 ± 10.2

Data are expressed as mean ± SEM. Normalized rCBF dropped to less than 30% during transient MCAO and returned to more than 70% at the onset of reperfusion. There was no statistically significant difference between the control and MEA groups at the same time points (*P* > 0.05).

**Table 4:** Normalized regional cerebral blood flow (rCBF, %) at different time points in relation to the onset of transient MCAO with MEA pretreatment in the study on western blot in the right cerebral hemisphere of the rats.

Group (number of rats)	Before MCAO	Onset of MCAO	30 min after MCAO	60 min after MCAO	Onset of reperfusion	30 min after reperfusion
Control (3)	100	23.2 ± 2.2	24.8 ± 2.6	26.2 ± 2.0	105.7 ± 6.6	106.4 ± 8.8
MEA (3)	100	25.2 ± 2.6	25.7 ± 2.5	27.6 ± 2.8	107.5 ± 9.1	114.9 ± 11.1

Data are expressed as mean ± SEM. Normalized rCBF dropped to less than 30% during transient MCAO and returned to more than 70% at the onset of reperfusion. There was no statistically significant difference between the control and MEA groups at the same time points (*P* > 0.05).
